# Bioacoustics as a Measure of Population Size and Breeding Success of European Storm Petrels *Hydrobates pelagicus*

**DOI:** 10.1002/ece3.71893

**Published:** 2025-08-11

**Authors:** Sophie Bennett, Lucy Williamson, Miguel Hernández‐González, Tom Denton, Rob Laber, Zoe Deakin, Mark Bolton, Ethan Manilow, Linda J. Wilson

**Affiliations:** ^1^ RSPB Centre for Conservation Science The Lodge Bedfordshire UK; ^2^ British Trust for Ornithology Scotland Stirling University Innovation Park Stirling UK; ^3^ Google LLC San Francisco USA

**Keywords:** population density, Procellariiformes, productivity, remote monitoring, remote sensing

## Abstract

Obtaining measures of population size and fitness are key first steps to understanding how and why species' populations change over time. Quantifying such metrics is difficult in some species, however, due to their remote location and/or ecology, that is they may be widely dispersed or may not be readily monitored visually. As such, bioacoustic monitoring is increasingly used to monitor populations of such species, as in burrow‐nesting seabirds. However, while a growing number of studies successfully obtain measures of population size using bioacoustics, there are few that effectively quantify measures of population fitness, limiting the conservation value of this tool. Here, we investigated whether bioacoustics could yield indices of population size and a key population fitness measure, breeding success, comparable to those derived from observer‐based methods in a breeding population of a burrow‐nesting seabird, the European Storm Petrel, *Hydrobates pelagicus* , on Mousa, Shetland. We used AudioMoths (Open Acoustic Devices) to record storm petrel adults and chicks over a 12‐week period from June to August 2023 and concurrently undertook observer‐based surveys. We then used a classifier model to quantify the average nightly call rate of adults and chicks across the recording period as bioacoustic‐derived measures of population size and breeding success, respectively. We found that observer‐based and bioacoustic‐derived measures of population size were significantly positively related for the two types of adult calls. Further, we found that observer and acoustic measures of chick abundance had a significant positive relationship, and a weaker, yet still significant, relationship for breeding success. Consequently, we demonstrate the utility of bioacoustics to provide relative measures of population‐level parameters and provide recommendations for future research. Bioacoustic monitoring can provide a method to monitor colonies while requiring substantially less time in the field, and so may facilitate more regular and comprehensive monitoring at colonies of burrow‐nesting seabirds.

## Graphical abstract

Quantifying changes in population size and breeding success metrics may prove difficult in some species due to their remote location and/or ecology, as in burrow‐nesting seabirds. Here, we investigated whether bioacoustics could yield indices of population size and breeding success comparable to those derived from concurrent observer‐based methods in a breeding population of European Storm Petrels, *Hydrobates pelagicus* , on Mousa, Shetland. Following the development of acoustic classifiers, we found clear positive relationships between bioacoustic‐ and observer‐based measures of both population size, chick abundance, and breeding success, demonstrating the utility of bioacoustics to provide relative measures of both population‐level parameters in seabirds for the first time.

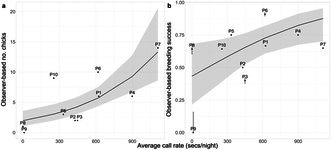

## 1 Introduction

Obtaining measures of population size and demographic rates is fundamental to understanding how populations change over time and identifying how they may respond to threats. The ecology of some species may make these data difficult to obtain; however, that is in the case of nocturnally active species or those that do not breed in open habitats (Buxton and Jones 2012; Keitt 2005). The collection of population‐level measures may also be time‐intensive and require multiple teams of observers in the field. Further, for remote populations of species, the collection of these data may be sufficiently logistically challenging that only very coarse and infrequent estimates may be obtained. As such, methods may instead prioritise obtaining measures of relative change as opposed to obtaining precise population measures. A number of techniques have been developed in recent decades that attempt to address these issues through remote sensing (Bird et al. 2022; Golightly and Schneider 2011; Lisovski et al. 2020). Bioacoustic monitoring is one such increasingly popular technique that has facilitated the collection of data for species that are not easily observed (Kelly et al. 2023; Oliver et al. 2018). As such, robust indices of population size and relative differences in population size within and between populations are being increasingly estimated using bioacoustic techniques, particularly in birds (Favaro et al. 2021; Kelly et al. 2023). However, there are currently limitations on the metrics that have been obtained using bioacoustics.

Bioacoustics have been effectively used to estimate an index of population density in a range of bird species (Francomano et al. 2024; Linares et al. 2022). Such bioacoustic indices may be quantified as the call rates of adults of either or both sexes, that is the frequency of occurrence or the duration of a call type per unit of time. Average call rates estimated over a number of days or nights can then provide comparative measures of changes in population size over time (Buxton and Jones 2012). Bioacoustics have also been shown to approximate other measures of population fitness, including breeding success (Schackwitz et al. 2020). Indeed, quantifying chick call rates using bioacoustics can be a more accurate indicator of breeding success than observer‐based techniques (Schackwitz et al. 2020). However, a key consideration with all such studies is that chick call rates may vary with satiation; such acoustic‐based measures must be interpreted with caution in many cases (Quillfeldt and Masello 2004; Ricklefs 1992). Despite the clear potential for bioacoustics to be used to obtain relative measures of breeding success, there are still few studies that investigate the potential utility of obtaining measures of breeding success using bioacoustics. As a result, there is at present a limit in the demographic parameters that may be measured using bioacoustics. Consequently, there is a need for studies investigating the potential of bioacoustic techniques to obtain demographic measures, particularly in those species where estimates of such measures currently prove challenging to obtain due to their ecology.

Burrow‐nesting procellariiform seabirds, such as petrels and shearwaters, are challenging species for which to obtain measures of population fitness. Such species tend to breed in single pair occupancy burrows that may be > 1 m deep underground (Sutherland and Dann 2012; Walsh et al. 1995). Additionally, most species of burrow‐nesting Procellariiformes are predominantly nocturnally active at the colony, and so are not easily observed (Winkler et al. 2020). The population size of such species may consequently be monitored using direct methods, where burrows are explored by hand and/or using endoscopes to confirm the presence of adults and/or young. As burrow‐nesting Procellariiformes are vocal at colonies, playback methods may also be used to estimate population size (Ratcliffe et al. 1998). For playback monitoring, recorded calls of the target species are played at sampling locations around a colony, and the response rate of adults calling back to the tape is measured and used to correct the observed density of responses to account for non‐responding individuals (Ratcliffe et al. 1998; Soanes et al. 2012). Both burrow inspection and playback are resource‐heavy techniques, however, and require considerable investments of both time and personnel and result in estimates with low precision (Bird et al. 2021; Soanes et al. 2012). Further, monitoring of productivity often requires multiple visits to nests throughout the breeding season, and/or expensive monitoring setups requiring purpose‐built monitoring chambers (Walsh et al. 1995). Obtaining measures of population size and breeding success in particular is also difficult in hard (or impossible) to access colonies of these species (i.e., those on steep cliffs and sea stacks). Given that breeding success may be higher in such inaccessible locations (e.g., due to lower vulnerability to disturbance or predation), observer‐based techniques may consequently under‐represent the full range of nesting habitats and breeding success. As a result, there are few examples for burrow‐nesting Procellariiformes of regularly recorded, high‐quality breeding success estimates. The limited and infrequent data that are collected on these species therefore limit our ability to understand their ecology, population dynamics, the impacts of threats, and the outcomes of conservation interventions.

Given the vocal at‐colony behaviour of many burrow‐nesting Procellariiformes (Robb et al. 2008), bioacoustics may present a less resource‐intensive and non‐invasive alternative to obtain measures of population size and breeding success in these species. Bioacoustic techniques have been successfully used to obtain an index of population size in some procellariiform seabirds through quantifying adult call rates (Golightly and Schneider 2011; Linares et al. 2022). However, relationships between call rates and measures of population fitness need to be established in a species before bioacoustics may be considered a viable alternative measure. Hence, there is currently a need for studies in a wider range of species. Further, bioacoustics have not been investigated as a method to obtain breeding success measures in any procellariiform species, thereby limiting the current utility of bioacoustic methods in monitoring procellariiform population fitness.

In this study, we investigate whether bioacoustics‐derived measures of population fitness (population size and breeding success) provide comparable indices to those derived from observer‐based methods. We undertook data collection on a population of European Storm Petrels, *Hydrobates pelagicus* , on the island of Mousa, Shetland. The presence of artificial nest boxes on the island facilitates easy access to a small number of nests, enabling studies of breeding biology and chick development, including how chick call rates change with age (Bolton 1996; Davis 1957). As adult storm petrels commonly elicit two types of calls (Robb et al. 2008), we investigate which of these calls, if any, is the most appropriate bioacoustic measure of population size (aim 1). We then estimate whether chick call rates change with age (aim 2) as a necessary prerequisite to investigating whether the call rate of storm petrel chicks may be used as an index of the abundance of chicks, and whether the call rate of storm petrel chicks may be used as indices of the abundance of chicks and/or observer‐based breeding success (aim 3a). We also test whether an entirely autonomous bioacoustic metric of breeding success derived from the acoustic data (chick call rate/adult call rate) relates to observer‐based breeding success (aim 3b).

## 2 Methods

### 2.1 Study Site and Species

The data in this study were collected from early June to late August 2023 on the island of Mousa, Shetland Isles, Scotland (60°00′N, 01°11′W). Mousa holds the largest population of storm petrels in the UK, numbering 10,778 breeding pairs (95% confidence interval: 8857–13,207) at the time of the most recent published whole‐island census in 2015 (Bolton et al. 2017). As such, storm petrels are protected under both the Special Protection Area (SPA) and Site of Scientific Interest (SSSI) designations of the island (work undertaken under a licence granted from NatureScot). The breeding population is distributed across the island, predominantly in boulder beaches, rock scree, and in disused buildings and structures, that is an Iron Age broch and stone walls.

Storm petrels rear a maximum of one chick in a breeding season and lay their single egg clutch from ~ mid‐June onwards in the UK (Davis 1957). The incubation period lasts c. 41 days (Davis 1957; Scott 1970) and eggs are incubated alternately by both parents. Chicks then remain in the nest for a further c. 65 days before fledging (Davis 1957; Scott 1970).

**FIGURE 1:**
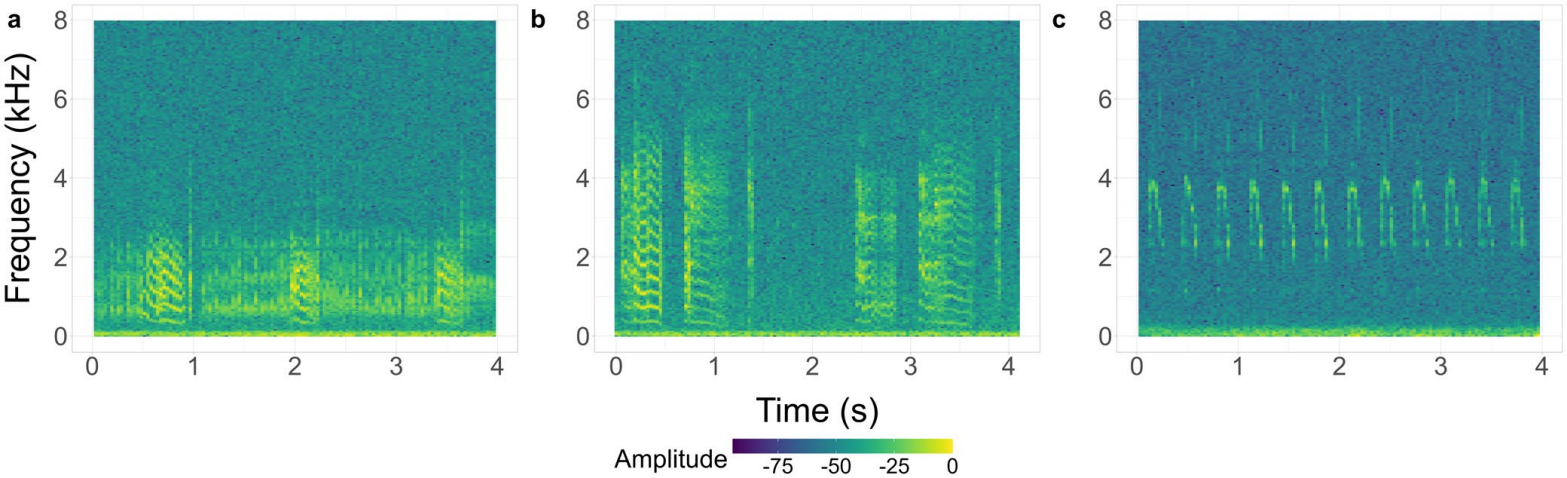
Frequency spectrograms of the three European Storm Petrel call types of interest: (a) adult: Purr, (b) adult: Chat, and (c) chick calls. Colour indicates amplitude measured in decibels.

Storm petrels may produce a range of call types depending on their age and sex (Robb et al. 2008). The most numerous and consistent of adult calls are'purr' and'cha', and for chicks,'beggin' calls. See Table 1 and Figure 1 for the features of these call types.

**TABLE 1:** Call types made by European storm petrels of differing age classes and sexes. Acoustic parameters of these call types are also provided, as derived from this study.

Age class	Call type	Sex	Frequency range (kHz)	Duration range (s)	Number of syllables	Inter‐syllable gap
Adult	Purr	Males	0.3–3	1–2	2	< 0.05
Adult	Chat	Females & males	0.2–6	0.8–1.5	3	0.1–0.3
Chick	Beg	Females & males	2.6–4	0.2–0.5	1	n/a

### 2.2 Study Design

**FIGURE 2:**
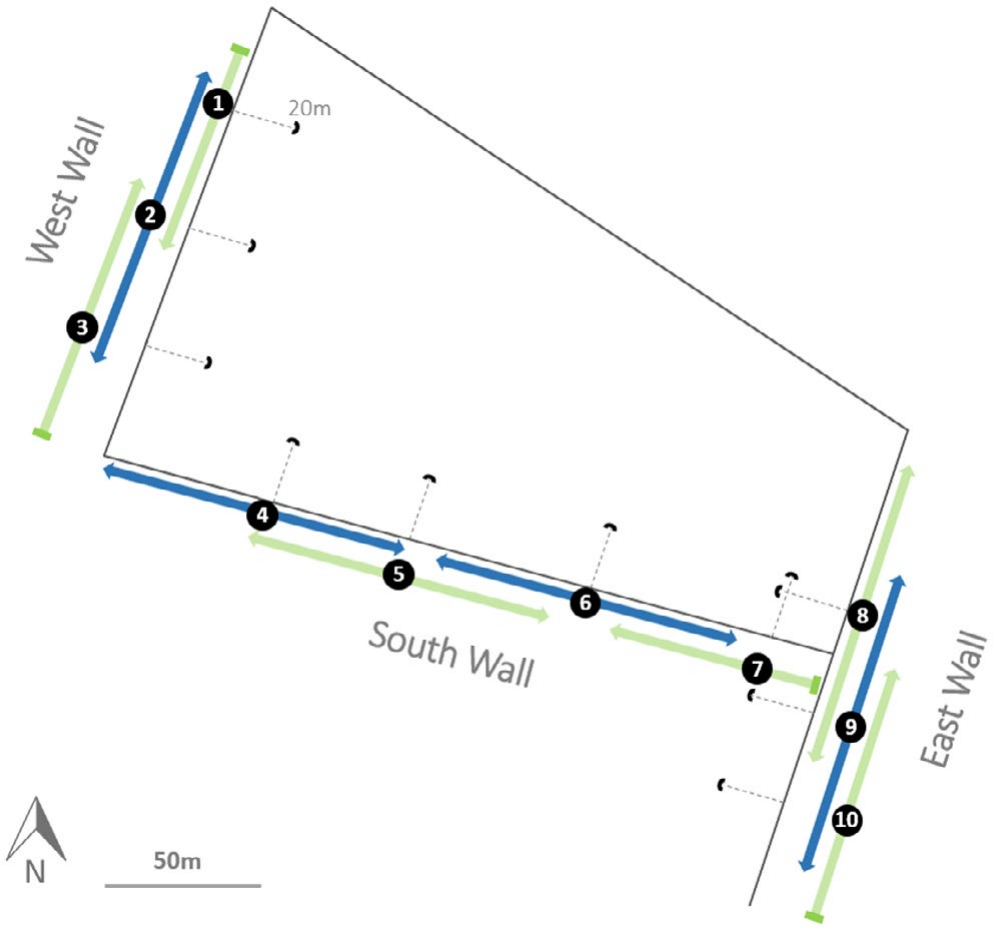
The relative locations of the 10 study plots on Mousa. Dotted lines indicate the line of sight of the receiver in each plot. Arrows indicate the extent of the detection tests undertaken in each plot along each wall. The standard extent of detection tests in plots was 50 m on either side of the plot centre; any deviations from this distance are indicated by flat‐ended arrows. A 50 m scale bar and compass direction are provided for context. All distances are to scale.

To compare both observer‐based and bioacoustic methods of deriving population size and breeding success, we undertook both approaches across 10 study plots located in stone walls in the centre of the island. See Figure 2. The storm petrels on Mousa do not nest in the grassy open areas on either side of the walls within the study areas, as there are few cavities for nesting and as this area is heavily trampled by the sheep stocked on the island. As such, we were confident that limiting the playback surveys to the walls themselves did not overlook any areas with breeding birds.

To record storm petrel calls, we used AudioMoths v1.2.0 connected to an external Clippy EM272 Mono lapel microphone. AudioMoths were placed in waterproof boxes with a small hole cut to allow the external microphone to be connected. The holes in the boxes were sealed with mouldable glue or putty, and a silica gel packet was placed inside to reduce any potential moisture damage to the devices. External microphones were fitted with foam covers to reduce the level of wind noise in recordings. The external microphones were secured in the centre of the base of a metal bowl acting as a parabolic receiver (28 cm diameter and 13 cm depth). The microphone and AudioMoth were secured to wooden posts at a height of 1 m and installed around a series of walls at a distance of 20 m perpendicular to the wall (Figure 2). Preliminary tests showed that placing parabolic dishes closer to the walls greatly reduced the length of the wall from which calls were detected (see below).

We made three visits to the island to collect the data for this study, spanning incubation to mid‐chick‐rearing. During the first visit (7th—20th June, i.e., early incubation) we installed the bioacoustic setup and undertook an observer‐based population survey of the plots. We serviced the AudioMoths on the second visit (9th—13th July, i.e., mid‐late incubation), where we charged batteries and downloaded data. On the third visit (17th—29th August, i.e., early‐mid chick‐rearing) we undertook observer‐based monitoring of breeding success. We downloaded the AudioMoth data and removed all equipment at the end of this final visit.

### 2.3 Bioacoustic Method: Detection Range Tests

To inform our main analyses, we first undertook tests to determine the length of the wall over which the receiver may detect the target calls of interest; an essential component of any bioacoustics study (Pérez‐Granados and Traba 2021). Undertaking these detection range tests informed the appropriate distance along the wall from each receiver over which to estimate observer‐based measures. Therefore, the detection range was not measured from the receiver, but rather it was measured along the wall from the plot centre, with the plot centre being the point on the wall directly opposite the receiver. For detection range tests, we played recordings of the storm petrel calls of interest throughout the length of each plot at 2 m intervals and recorded the range in which calls were audible in the downloaded recordings from the AudioMoths. that is calls were played every 2 m along the green and blue arrows in Figure 2.

We determined the average detection range (to the nearest 2 m either side of the plot centre) of each call type across plots to be 26 m (95% confidence interval (95% CI) = 18–38), 30 m (95% CI = 22–42), and 16 m (95% CI = 10–20) for the adult purr, adult chat and chick calls, respectively. See Data S1 for a detailed description of these tests. Consequently, the detectable range of each plot did not overlap with one another for chick calls, but there was a small degree of overlap for both of the adult calls (*n* = 7/129; 5.4% nests overlapped with two plots). Given the small proportion of nests that would have then been recorded by more than one recorder, and that the maximum number of nests that overlapped from a single plot was three, we think it unlikely that this would have caused any clear bias in our results.

### 2.4 Bioacoustic Method: AudioMoth Configuration and Recording Period

We programmed AudioMoths to record from 2200 until 0200 for each night of the study period, in each plot (all times in Greenwich Mean Time, GMT). We chose this recording period as the acoustic activity in storm petrels predominantly takes place in near‐full darkness (Collins 2021), which was limited to between 2200 and 0200 for the study site in much of the study period. AudioMoths were configured with a sampling rate of 16 kHz and with gain set to'medium'. We split the recordings into two groups: one to estimate adult call rates prior to hatching, and one post‐hatching in which to estimate chick call rates. We divided the recordings into these two groups on 14th July, 1 week prior to the estimated first hatch date for chicks in the study plot. (We estimated the ages of chicks from their tarsus length measurements as per Bennett and Bolton (2025) *In review* and the oldest chick in a nest box was estimated to have hatched on the 21st of July). We did not include post‐hatching recordings when estimating adult call rates as the number of non‐breeding birds calling can increase at this time of the breeding season (Ratcliffe et al. 1998), and breeding birds may begin to leave chicks unattended to forage (Scott 1970). We included a further buffer period of a week to account for earlier‐hatching chicks and known errors in estimating chick ages.

The range of dates included in the analysis for each plot varied due to the time taken to install and remove devices, as per Data S1. Prior to analysis, we filtered out those recordings with unusable data based on the occurrence of disruptive background noise, such as high wind speeds (Data S1). Storm petrels may have significantly decreased activity during full moons as a predator avoidance strategy (Watanuki 1986) so we also filtered out those data from nights when the moon was > 75% full and vocal activity rates were expected to be less consistent (Oppel et al. 2014; Raine et al. 2017). Ultimately, we collected an average of 29.1 ± 3.57 and 33.6 ± 5.32 nights (mean ± standard deviation) of usable data across all plots for adult and chick calls, respectively.

### 2.5 Bioacoustic Method: Quantifying Storm Petrel Call Rates

To obtain estimates of adult and chick call rates, we ran all recordings through an acoustic classifier model via the package'perch', available via GitHub (Google LLC 2024). Attempting to train a new deep learning model from scratch for a niche use case, such as differentiating call types within a single species, is often prohibitively expensive in terms of computing resources and annotated data. To address this, we employed a technique called Agile Modelling (Stretcu et al. 2023) that leveraged embedding vectors (a set of vectors containing information on acoustic properties of recordings that the model uses to undertake classification) created by a pre‐trained deep learning model that was trained for bird call identification (Ghani et al. 2023). Here,'leveraging’' means generating these embeddings that distinguish between different call types. The Agile Modelling workflow involves a step akin to'bootstrapping’' whereby a similarity search for unlabelled audio snippets that have an embedding vector that is close in space to the vector corresponding to a known (i.e., labelled) audio snippet, that is audio snippets that are structurally similar to and close in time to labelled snippets are given the same label, is used to increase the effective sample size of labelled audio snippets. The'bootstrapped' labelled dataset can then be used to train a simple linear model over the embedding space.

We employed this method by using a pre‐built birdsong classification model (Google Research 2023) to generate the embeddings for our unlabelled audio dataset. We then generated a training set consisting of examples from that dataset by performing several iterative nearest‐neighbour searches using a kNN (k‐nearest neighbours) search. The kNN search across the corresponding embeddings used known examples of the desired call types (‘adult: purr’ = five examples; ‘adult: chat’ = five examples; ‘chick’ = eleven examples) and manually listening to and annotating the results. We included a larger number of chick call type examples (eleven) as there was greater variability in this call type, associated with chick growth. The resulting dataset was then used to train a simple linear model over the embeddings for each call type, which we then ran over the entire unlabelled dataset to generate cumulative statistics for the entire audio dataset, including the number of instances of each call type that were detected. In this model, the explanatory/independent variable is a single 1280‐dimensional vector representing the embedded 5‐s audio sample, and the response variable is a vector of length 4 whose values represent the model's logit response for each of the three target call types, and a fourth category for all non‐target calls. Model performance was assessed by (a) overall accuracy, which is the percentage of correct top‐1 (i.e., whether the class with the highest probability was the same as the target label) predictions from the model, (b) ‘Area Under the Curve of the Receiver Operating Characteristic’ (AUC‐ROC), which is a measure of model robustness, and (c) Mean Average Precision (mAP), which summarises the per‐class model performance. For the full modelling process, see Data S1 and Ghani et al. (2023).

Finally, we estimated nightly call rates from the output of the classifier as the length of time in seconds that each of the three target call types was recorded in each plot each night. To estimate a mean call rate, we then calculated the average nightly call rate in each plot across the study period for the three call types (adult purr, adult chat and chick).

### 2.6 Bioacoustic Monitoring Method: Quantifying Variation in Storm Petrel Chick Call Rate With Age

We undertook additional tests to determine whether the call rate of storm petrel chicks was consistent, independent of their age, as per Quillfeldt (2002). A number of nest boxes have been installed in the stone walls on Mousa over the past 30 years; a small number within the survey area (Bolton 1996). We installed AudioMoths in 10 of these nest boxes that were occupied by at least one adult in June prior to laying (see Data S1 for nest box locations). These AudioMoths were recorded on the same schedule and settings as those in the detection array (i.e., nightly, 2200–0200 GMT). We then collected these AudioMoths in late August. Nine of the 10 AudioMoth‐containing nest boxes reached the chick stage of breeding. The recording period of the nest box AudioMoths spanned from pre‐hatching to mid‐chick‐rearing (Data S1).

### 2.7 Observer‐Based Monitoring Method: Population Size (Apparently Occupied Nests)

We used playback methodology, as per Ratcliffe et al. (1998) and Bolton et al. (2017) to obtain observer‐based measures of population size as the number of Apparently Occupied Nests (AONs) in each plot. We played a recording of adult purr calls for 10 s at 1 m intervals along the wall 50 m on either side of the centre of each plot. When birds responded to the call, we identified and marked the location of each AON with a unique identifier. Given that not all adults will respond to playback on any one occasion, we then repeated the survey seven times, once per day, in each plot. On subsequent visits, we recorded which previously marked and unmarked AONs responded to the playback. All playback was undertaken between 0900 and 1800 when response rates are most consistent (Ratcliffe et al. 1998).

We recorded the distance of every identified AON from the receiver in each plot with a laser range finder (to the nearest 1 m) so that we could identify those nests within the detection range of the receiver in each plot, as per the outcome of the detection range tests. This included any nests in walls adjacent to the plot that were within the detection range (Data S1). Consequently, for each plot, we included only those nests within audible distance of the receiver, and so within the detection range of the receiver in each plot.

We then estimated population size from the playback surveys as per Bolton et al. (2010) using the Bolton et al. (2019) Shiny App tool. The Shiny app tool estimates 95% confidence intervals of the population size and estimated response rates through a bootstrapping procedure with 999 replicates for the survey area in each plot. We then estimated the number of AONs that were likely present but undetected in the surveys using those derived nightly response rates as per the following formula:

$$\%\text{nests undetected}=100*{\left(1-\text{response rate}\right)}^{\text{number of trials}}$$

Where ‘response rate’ was the average proportion of nests that elicited a call across survey visits (seven) for each plot, estimated from the Shiny App tool (Bolton et al. 2019). For all plots, the ‘number of trials’ was seven. The estimated mean percentage of nests left undetected across plots was 9.6% ± 5.2% (mean ± standard deviation).

### 2.8 Observer‐Based Monitoring Method: Breeding Success

In August, we estimated the breeding success of those AONs identified in each plot. We first used endoscopes to identify the contents of each accessible nest site identified in the observer‐based population size playback surveys in June that were within the detectable range of receivers (128/129 nests identified in the playback survey were checked). We searched the marked location of each nest site for a maximum of 10 min, stopping if any adult, chick, or egg was observed. If no adult, chick, or egg was seen on the first search, we then searched the site a second time on the following day to confirm that the site was empty. Any empty sites or those with an unattended egg were considered unsuccessful (44/129 empty sites were unsuccessful, 1/129 with an unattended egg).

The abundance of larger, older chicks may be used to infer the breeding success of storm petrels (Bolton et al. 2010), as the majority of nesting failure occurs in the incubation, brooding, and early chick‐rearing stages (Minguez and Oro 2003). The tarsus length of chicks may be reliably used to age chicks up to ~30 days of age (Bolton 1995), at which point 96.7% of nest mortality is likely to have occurred (Minguez and Oro 2003).

To determine whether the chicks in the nests identified in this study were sufficiently advanced in their development that their presence indicated a nesting attempt that was highly likely to be successful, we measured the tarsi of and aged all accessible chicks that we encountered in the study area to the nearest 0.1 mm using callipers. From these tarsus measurements, we estimated the ages of chicks to the nearest ±1 days as per (Bennett and Bolton 2025). We found that the average age of chicks in the study plots at the time of surveying was 24 days post‐hatching (95% confidence interval = 23, 25 days). At 24 days post‐hatching, the chicks are highly likely to fledge, as 94.1% of nest failures occur by this point (Minguez and Oro 2003). So, we are confident that the presence of chicks in study plots at the time of surveying is indicative of successful breeding.

### 2.9 Statistical Analysis

All analysis was undertaken in the statistical software R version 4.2.2 (R Core Team 2023). All plots were produced using the R package'ggplot' (Kassambara 2019). We mean‐centred and standardised all continuous normally distributed variables prior to testing. We considered effects in models to be significant if their 95% confidence intervals did not cross zero. Means are presented ± standard deviation unless stated otherwise. As there was the potential for non‐linear relationships between call rates and population measures, for all models we tested whether a polynomial model (quadratic, cubic) produced a better model fit. We determined which of these polynomial models produced the best fit through comparison of AIC values and considered models with a lower AIC, where ΔAIC > 2, to be of better fit (Burnham and Anderson 2003).

#### 2.9.1 Aim 1: Bioacoustics as an Index of Observed‐Based Measures of Population Size

To test whether bioacoustic measures may be used as an index of population size, we first tested whether we had sufficient nights of data to obtain a stable and robust measure of call rate using an accumulation curve. We randomly selected nights without replacement and recalculated the average nightly call rate with the addition of each new night's data. We then tested whether the curve reached no significant change in the average call rate estimated, which we defined as a slope of < 0.1. For both adult call types, the nightly call rate reached a stable measure within the number of nights of data obtained (13 ± 8.18 nights, adult purr; 11.9 ± 9.29 nights, adult chat, see Data S1).

We then used a generalised linear model (GLM) to determine whether either adult call rate was a clear predictor of population size. None of the tested polynomial models produced a better fit (ΔAIC quadratic model = 6.4, cubic model = 10.3). The observer‐based measure of'population size' (number of AONs, accounting for non‐responding birds) was the response variable fitted with a Poisson distribution. The ‘call rate’ (average number of seconds per night) of adults was included as an explanatory effect. As adult storm petrels may commonly elicit chat calls or purrs, either call may be a suitable target call with which to measure population size. Thus, we ran two analyses, one with adult: purr and one with adult: chat as the explanatory effect. It was not appropriate to include both call types in one model for comparison of effect presence and strength because there was covariation between the two variables.

#### 2.9.2 Aim 2: To Quantify Any Changes in Chick Call Rate With Chick Age

To determine the appropriate range of dates to include for Aim 2 (i.e., from the date of hatching), we first back‐calculated the estimated ages of chicks in nest boxes with AudioMoths as per ‘Authors of current study’. We then quantified the nightly call rate for each chick (*n* = 9) for each day of available data from the estimated hatch date through to the last day of recording (see Data S1 for the recording periods for each nest box). We then used a general additive mixed model (GAMM) to investigate the relationship of chick call rate with age using the R package ‘mgcv’ (Wood 2011). We used a GAMM here as opposed to a GLMM, as we did not necessarily expect the change in chick call rate with age to be linear. ‘Chick call rate’ was the response variable with a Gaussian distribution and a log‐link. ‘Chick age’ in days was the only explanatory variable, and ‘chick ID’ was included as a random effect to account for individual‐level variation in call rates. We specified the number of knots at eight. We also include an auto‐regressive term for ‘night’ to account for any temporal autocorrelation in call rates due to extrinsic factors that may affect either call rates or the amount of background noise in recordings.

#### 2.9.3 Aim 3: Bioacoustics as a Predictor of Observer‐Based Measures of Chick Abundance and Breeding Success

We tested the relationship between the bioacoustic and observer‐based measures of chick abundance and breeding success. Given that we did not necessarily expect chick call rates to be constant over time, as this may change with satiation and chick age, we did not require chick call rates to reach stability. However, for reference and comparison, as for the adult call types, we calculated the minimum number of nights of data needed to reach a plateau for chick call rate (average 25 ± 9.19; Data S1). In only four plots was a stable plateau of chick call rate reached within their recording period. As variation in We used GLMs to test the relationship between observer‐based assessment of both the total number of chicks per plot, and breeding success (number of chicks found during burrow scope searches/number of breeding attempts, i.e., the number of nests that were identified as occupied during the playback surveys) and the call rate of chicks (aim 3a). In the first model, the ‘number of chicks’ was the response variable and was fitted with a Poisson distribution. In the second model, ‘breeding success’ was the response fitted as the number of successes (i.e., chicks still present when assessed using observer‐based measures) and failures (i.e., chicks not present when assessed using observer‐based measures) as a binomial distribution with a logit link. In both models ‘chick call rate’ was the only explanatory variable. For the first model, where the number of chicks in a plot was the response, none of the tested polynomial models produced a better fit than the linear model (ΔAIC quadratic model = 8.5, cubic model = 21.5). For the second model, where the breeding success of a plot was the response, the quadratic model produced the best fit (ΔAIC linear model = 13.4, cubic model = 16.7). Lastly, to test aim 3b, that is whether an acoustic measure of breeding success estimated in the same manner as an observer‐based measure of breeding success (i.e., measure of chick abundance/measure of breeding pairs) is related to that derived from observer‐based methods. In this model, ‘observer‐based breeding success’ was again the binomial response variable with a logit link. The single explanatory variable was the ‘chick call rate’ in each plot divided by the adult call rate. We ran one model where the ‘chick call rate’ was divided by the adult: purr call rate, and another by the adult: chat call rate. In both models, the quadratic produced the best fit (ΔAIC linear model = 5.4, cubic model = 6.1). Only the models with the best fit are presented in the results.

## 3 Results

### 3.1 Aim 1: Bioacoustics vs. Observed‐Based Measures of Population Size

**FIGURE 3:**
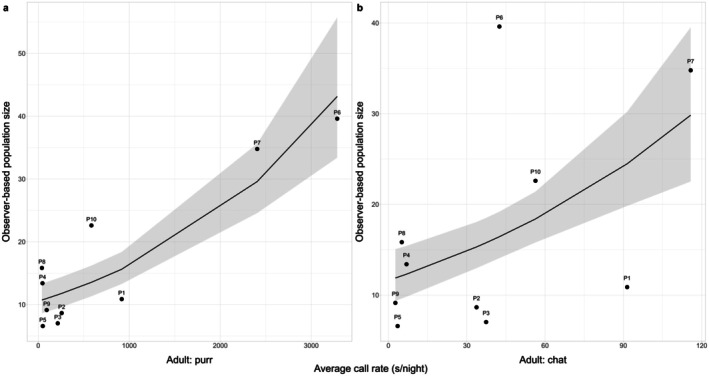
GLM predictions of the relationship between bioacoustic and observer‐based measures of population size for (a) Adult: Purr calls (black line), and (b) Adult: Chat calls. Error bars indicating the standard error in population size estimates for each plot across the possible detection range along the wall are not provided as these were too small to be seen in the figure. *n* = 10 in both plots. *R* ^2^ values: (a) 0.80, (b) 0.63.

There was a strong positive relationship between the observer‐based measure of population size and the call rate of adults for the purr call type (estimate = 0.49, 95% CI = 0.36, 0.61), and a weaker, but still significant effect, evident for the chat call type (estimate = 0.32, 95% CI = 0.17, 0.46). For both call types, the confidence intervals around the relationship between call rate and observer‐based population size widened as both values increased (Figure 3).

The adult: purr and adult: chat call rates of plots were proportionally similar to one another, but not significantly correlated (*p* = 0.07).

### 3.2 Aim 2: Changes in Chick Call Rate With Chick Age

**FIGURE 4:**
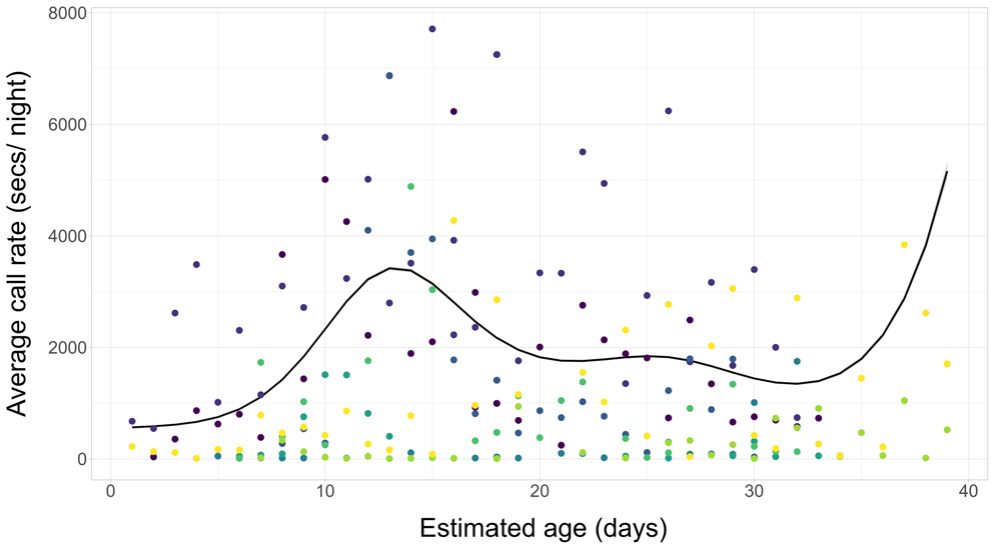
GAMM predictions of the relationship between storm petrel chick age and the nightly call rate of chicks (black line) with 95% confidence intervals (shaded area). Raw data is provided as points, where colour indicates individual chick ID. *n* = 218.

Chicks raised in nest boxes started to call for the first time from 0 to 5 days post‐hatching, and call rate increased throughout the first 2 weeks post‐hatching (Figure 4). After the first 2 weeks, the call rate fell slightly but remained broadly constant thereafter (Figure 4). From c. day 30, there was a further increase in the call rate; however, these data largely arose from just two individuals with few data points and so may be less representative of the population‐level relationship.

The random terms of night and chick age explained a large proportion of model variance, indicating that there was both clear temporal and individual‐level variation in the relationship between call rate and age (*R* ^2^ marginal = 0.14, *R* ^2^ conditional = 0.58).

### 3.3 Aim 3: Bioacoustics vs. Observer‐Based Measures of Chick Abundance and Breeding Success

**FIGURE 5:**
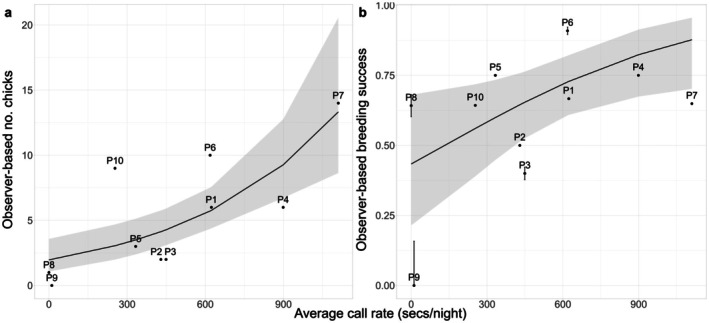
GLM predictions of the relationship between bioacoustic and observer‐based measures of (a) the number of chicks, and (b) breeding success (black lines). In (b), error bars indicate the standard error in breeding success estimates for each plot across the possible detection range along the wall, though these are generally too small to be seen in the figure. *n* = 10. *R* ^2^ values: (a) 0.56, (b) 0.42.

The chick call rate in a plot had a significant positive relationship with the number of chicks detected using observer‐based methods and, to a lesser extent, with the overall breeding success in a plot, as detected using observer‐based methods (number of chicks: estimate = 0.61, 95% CI = 0.33, 0.90; breeding success estimate = 0.71, 95% CI = 0.14, 1.35; Figure 5).

The number of chicks and observer‐based breeding success were also highly correlated with one another (cor = 0.80_df=8_, *p* < 0.01).

When testing aim 3b, we found no relationship between observer‐based breeding success and the autonomous metric of acoustic breeding success (chick call rate/adult call rate) for either adult call type (model with adult: purr: 95% confidence interval = −0.34, 0.76; adult: chat: 95% confidence interval = −0.28, 0.92).

## 4 Discussion

We demonstrate that the call rate of adult storm petrels had a significant positive relationship with population size. We show for the first time in a procellariform seabird that the call rate of chicks has a significant positive relationship with the abundance of chicks; however, we found no relationship between the acoustic measure of breeding success (chick call rate/adult call rate) and observer‐based breeding success, likely due to differences in the detection range of calls of adults and chicks. We also document in detail for the first time how the call rate of storm petrel chicks may change over the first few weeks of growth. Importantly, we show that storm petrel chicks may begin emitting audible calls from the first day post‐hatching. Overall, we show that bioacoustic measures of population size may be used as a comparative index for this species that may be used to monitor changes in these measures over time.

Our results show that an average nightly call rate of adult storm petrels could be used as an index of population size. Our results align with those in Leach's Storm Petrel (Golightly and Schneider 2011), Streaked Shearwater *Calonectris leucomelas* (Hart et al. 2021) and Cory's Shearwater (Oppel et al. 2014) that demonstrate that the mean nightly call rate captured over a sufficiently long period can be used as an index of population size. However, given the much greater deviation of raw points from the model predictions for the adult‐chat calls, we would not recommend that chat calls be used to detect relative changes in population size of storm petrels. The scale of difference in population size that we detect using nightly call rate is relatively small in terms of the number of nests, from the smallest plots of ~10 breeding pairs up to ~30 breeding pairs. Further, the relative population sizes in this study are within the range of those in Oppel et al. (2014) (mean population size = 19.89 ± 5 nests; population size not provided in Golightly and Schneider 2011). As with Oppel et al. (2014), however, the imprecision of the bioacoustics method to detect exact changes in population size results in a range of potential population sizes as opposed to a single absolute value. A relatively large degree of imprecision of burrow‐nesting seabird population counts is common to all currently used methods due to the species' ecology (Soanes et al. 2012). Bioacoustics could nonetheless provide an option for more regular monitoring of colonies due to the reduced resources required in the long term (after the initial purchasing of equipment). Due to the lower requirement for resources for long‐term monitoring, bioacoustics could be a more affordable and time‐efficient option to detect change in populations than currently used methods that require substantially more time in the field. Further, detecting small changes in population size (i.e., where the change is small either in absolute numbers of breeding pairs or as a proportion of the total population) is a challenge for any monitoring technique, in part due to imperfect detection of all nests. In part, the difficulty in obtaining a reliable call rate for smaller populations may arise from the increased sample size of nights that is required to estimate a stable call rate. There may also be a point at which recordings are completely saturated with calls, and so any further increases in population size could not be measured. Further data collection comparing these methods would be required from a more broadly distributed sample of population sizes to increase the confidence in the power of bioacoustic methods to monitor population size.

Stone walls are not the only habitat on the island used by storm petrels; the birds may also breed in the stone buildings on the island, boulder fields, and boulder beaches. However, the stone walls were the only habitat type in which we could be confident of detecting the majority of the nests using the observer‐based methodology. Consequently, we consider only the stone wall habitat type here, but we would expect our results to be broadly applicable to other storm petrel habitat types where nests fall within the detection range of the recording equipment used. To understand how acoustic methods perform in two‐ and three‐dimensional habitats (such as boulder beaches) where the distances of nests from the recorder vary considerably more than in the one‐dimensional habitat we studied, we recommend additional testing in these habitats to confirm reproducibility. Overall, our results provide evidence that bioacoustic monitoring of storm petrels may be used to estimate relative population size in breeding colonies that are inaccessible to observers but are within the detection range of audio equipment. As such, bioacoustic monitoring may facilitate more frequent monitoring of previously unexplored and/or inaccessible colonies (e.g., those on sea stacks and sheer cliffs), if sufficient quality recordings can be obtained. In those colonies that are accessible and more easily monitored currently, continued comparisons and ground‐truthing of the two methodologies would be prudent.

Interestingly, we find that a stable adult call rate, for both purr and chat calls, was achieved with fewer nights than those recorded in Buxton and Jones (2012) on a taxonomically similar species, the Leach's Storm Petrel *Hydrobates leucorhous* (*n* = 75–97.1 nights). In contrast, for the most similar call type in this study (adult: chat calls) we identified a plateau after 11.9 ± 9.29 nights. The large difference in the number of nights of data required to obtain a stable measure of call rate here may result from a clear difference in the call rates in both studies and from the clear difference in the structure and form of the calls in both species. In Buxton and Jones (2012) the majority of colonies where call rates were quantified did not exceed 7 calls/night, in contrast to the ~40 s of chat calls/night in our study, which equates to a minimum of 26 calls/night, assuming that all calls had the maximum observed length of 1.5 s. For adult purr calls, we find an average call rate in excess of 200 s/night. Buxton and Jones (2012) also found a trend of colonies with higher call rates reaching a plateau in fewer nights due to a more stable call rate in such colonies. The rationale of'noisier’' colonies reaching a plateau sooner would then support the far smaller number of nights required to reach a stable call rate in this study. We further show that the number of nights required to reach a plateau varied between call types. This variation may arise from the differing biological function of the call types: purr calls are made by territorial males; chat calls are made by breeding individuals of both sexes (Robb et al. 2008). The variation between call types and study systems in the number of nights of data required to reach a plateau indicates that the minimum required number of nights per recording period will vary between species and colonies. Importantly, in this study, we demonstrated that more than 3 weeks of recording were required to obtain a stable call rate across call types. As such, future studies must endeavour to obtain sufficient nights of data to obtain stable call rates for analysis, and this may vary with the acoustic activity at the colony level. With that, we would recommend that in the first instance, studies begin by recording for at least 30 nights over an entire lunar cycle when monitoring population size.

We show that relative differences in bioacoustic measures of chick call rates relate to relative differences in intensive observer‐based measures of chick abundance and, to a lesser extent, breeding success. However, we found no relationship between the autonomous acoustic measure of breeding success and observer‐based breeding success, that is measure of breeding pair abundance/chick abundance. To our knowledge, this study is the first to show that bioacoustics may be used to infer comparative differences in measures of seabird chick abundance and breeding success, and therefore longitudinal changes in breeding success in Procellariiformes. There are, however, other studies that may show a relationship between call rate and breeding success in other bird families (Schackwitz et al. 2020) or simply between call rate and the number of chicks (Francomano et al. 2024). Despite the clear need for new techniques to monitor breeding success in Procellariiformes this area is rather neglected at present. Obtaining observer‐based measures for breeding success often requires more time and resources than obtaining measures of population size alone for birds, due to the requirement for repeated visits to a colony to establish whether chicks fledge successfully (Walsh et al. 1995). As a result, as we demonstrate, the adoption of a bioacoustic‐based monitoring method requiring only one trip to install recorders and a second to retrieve them may provide a less resource‐intensive method to obtain a comparative measure of breeding success. The practical benefits of using bioacoustics to monitor breeding success have been further acknowledged by similar recent studies (Francomano et al. 2024; Golightly and Schneider 2011). As breeding success is derived ultimately from the number of chicks divided by the number of AONs in a plot, it is not surprising that the relationship between chick call rate is related more strongly to the number of chicks present rather than breeding success. Indeed, the relationship we observed may arise from a fortunate year in which the number of chicks related to breeding success (i.e., the majority of chicks survived to fledging), which is not necessarily always the case. Crucially, then, it is important that future studies investigating the utility of bioacoustics to monitor burrow‐nesting Procellariiformes do so over a range of years, sites, and environmental conditions experienced by the birds to increase confidence in the true robustness of this technique. The relationship between chick call rate and breeding success was weaker than that with chick abundance; however, this does indicate that caution and careful consideration of how to interpret bioacoustic‐derived measures of breeding success must be observed. The lack of a stronger relationship between chick call rate and observer‐based breeding success was exemplified by the lack of a relationship between the autonomous acoustic measure of breeding success and observer‐based breeding success. A potential cause for this lack of effect is that chick and adult calls were capable of being detected over different ranges; chick call rates could be detected from c. two‐thirds of the range of adults. Consequently, the two bioacoustic‐based measures are not equal samples as the equivalent observer‐based measures are. Another consideration is that in this study, it was not possible to leave recorders in place through to the end of fledging for chicks. There may then be a more optimum period in chick growth to test the relationship between chick call rates and observer‐based breeding success. Investigating this latter point through resampling or using recordings from varying periods in chick growth, in particular, should be a focus of future research in this area. Given that we find a significant relationship between two observer‐based measures and chick call rate, with a relatively small number of plots, indicates the potential power of bioacoustic methods to monitor storm petrel populations. Clearly, though, using the methodology in this study, bioacoustic‐derived measures of breeding success cannot be obtained in the same manner as those of observer‐based methods (i.e., chick abundance measure/breeding pairs measure). Importantly, we also demonstrate that European storm petrels may call from their first day post‐hatching; earlier than the c. 10 days of age identified by Scott (1970). As a result, bioacoustics may also prove a useful measure to monitor hatching dates in this species on an individual level, where recorders are placed in a nest box, or provide a coarser population‐level measure of breeding timing, where recorders are placed within colonies.

We further demonstrate that bioacoustics can be used to update and improve our understanding of the basic ecologies of well‐studied species. We found no clear overall relationship between chick call rate and age, as also observed in Wilson's Storm Petrel *Oceanites oceanicus* (Gladbach et al. 2009). There are several studies finding relationships between a chick's hunger and their call rate (or ‘begging rate’) (Klenova 2015; Quillfeldt and Masello 2004; Ricklefs 1992). In years when prey conditions are less favourable, chick call rates may increase or conversely decrease in years of more favourable conditions, irrespective of the number of chicks present in a colony. Given that the breeding season in the study year was better than average (overall breeding success estimated at 75%, or 0.75 fledged chicks/apparently occupied nest), the chicks may well have been largely satiated in this study, allowing an age‐independent effect of call rate with age to be elucidated. Interestingly, we found that more days of recording data were required to reach a stable call rate for chicks (20.5 days on average) compared to either adult call type (adult: purr *n* = 75–97.1 nights, adult: chat 11.9 ± 9.29). This may arise from the general greater variability in the chick call rates, and that chick call rates may vary with, for example differing hunger levels, as discussed here. Clearly, there is considerable scope for future studies to further investigate the calling behaviour of storm petrel chicks in relation to age and potentially also local food supplies. A further consideration is that the timing of hatching of chicks in nest boxes in this study was broadly synchronous. In years, colonies, or species where breeding timing may be less synchronous, there may be subsequent effects on the utility of bioacoustics to monitor breeding success, as many older, larger, potentially louder chicks beyond the age tested in this study may be calling concurrent with many younger, smaller, and quieter chicks, so affecting derived call rates.

Both observer‐based and acoustic‐based measures of population fitness are subject to a set of biases. Observer‐based measures of population size will be subject to methodological limitations, many of which are the same for bioacoustics (e.g., resulting from imperfect detection rates). As a result of their set of biases, either method may be more appropriate to use in certain species, resulting from their behaviour and breeding biology, or certain colonies (e.g., due to differences in accessibility). It should also be considered that the detection ranges quoted in this study will be specific to the exact setup used here, and it is imperative that all studies attempting to use bioacoustics to monitor populations in an analogous manner quantify the on‐site detection ranges of their acoustic receivers. Following this, we would recommend that the relationships between call rates and population fitness measures be re‐tested in each new species and variation in recording equipment setup prior to being the sole technique used. Lastly, the actual number of breeding pairs and, subsequently, chicks that we monitor in this study is relatively small, as was the number of plots for which we could obtain data. We collected data across ten experimental study plots, which, while relatively few, were the maximum that logistics allowed. Despite this limited sample size, however, we demonstrate relationships with relatively small errors between observer and bioacoustic‐derived measures of population size and breeding success that were unlikely to be present in the absence of a'tru' effect. The errors associated with the relationship for population size were the largest for the relationships modelled and are likely to have resulted from the limited number of plots from the medium population sizes tested. Consequently, we would recommend that future studies be undertaken across the wider range of possible population densities of this species to refine this relationship. Additionally, in this study, there were never incidences of the maximum potential call rate being reached (i.e., calls for the entire duration of all 4 h of nightly recording time), either on an individual night or a derived mean call rate. In populations that are sufficiently large and/or dense, and with a very high level of bioacoustic activity, the maximum possible call rate will be reached. In these sufficiently large colonies, it will no longer be possible to detect further increases in population size using the techniques described in this study.

Overall, we demonstrate a positive relationship between the call rate of adult storm petrels and population size, and between chick call rate and both the number of chicks and breeding success, and provide detailed information on the call rate of storm petrel chicks for the first time. As such, we find evidence to suggest that bioacoustics‐derived measures of call rates may prove to be reliable relative indices of population fitness. However, given the broader confidence intervals associated with estimating changes in population size at larger population sizes, we recommend that additional studies be undertaken that include the full range of possible population sizes for storm petrels. We recommend that adult purr calls be used in future studies to estimate relative changes in population size, and chick calls for the abundance of chicks and breeding success of storm petrels. We recommend that those wishing to undertake bioacoustic surveys of burrow‐nesting seabirds undertake similar detection range tests as we describe here. Furthermore, we recommend that they determine, if not already robustly tested, whether bioacoustic measures of fitness relate to those observer‐based methods currently used and, importantly, include the likely error associated with any quantified fitness measure to ensure that overconfidence in bioacoustic‐based measures is not stated. This last point we would recommend for all methodologies used to monitor these populations, as further recommended by (Bird et al. 2021). Future investigations into what other population measures and species behaviour may be elucidated from bioacoustic methods may facilitate an improved understanding of the fitness of burrow‐nesting seabird species populations.

## Author Contributions

**Sophie Bennett:** conceptualization (equal), data curation (lead), formal analysis (lead), investigation (lead), methodology (equal), project administration (equal), supervision (lead), visualization (lead), writing – original draft (lead), writing – review and editing (equal). **Lucy Williamson:** data curation (equal), writing – review and editing (equal). **Miguel Hernández‐González:** data curation (equal), writing – review and editing (equal). **Tom Denton:** methodology (equal), writing – review and editing (equal). **Rob Laber:** methodology (equal), writing – review and editing (equal). **Zoe Deakin:** methodology (equal), writing – review and editing (equal). **Mark Bolton:** conceptualization (equal), methodology (equal), project administration (equal), visualization (equal), writing – review and editing (equal). **Ethan Manilow:** methodology (equal), writing – review and editing (equal). **Linda J. Wilson:** conceptualization (equal), investigation (equal), methodology (equal), project administration (equal), visualization (equal), writing – review and editing (equal).

## Conflicts of Interest

The authors declare no conflicts of interest.

## Supporting information


Data S1.

## Data Availability

The processed data and code are provided as Supporting Information to this manuscript.

## References

[ece371893-bib-0001] Bennett, S. , and M. Bolton . 2025. “Tarsus Length as a Predictor of Chick Age in European Storm Petrels *Hydrobates pelagicus*.” In Review.

[ece371893-bib-0002] Bird, J. P. , R. A. Fuller , P. P. Pascoe , and J. D. S. Shaw . 2022. “Trialling Camera Traps to Determine Occupancy and Breeding in Burrowing Seabirds.” Remote Sensing in Ecology and Conservation 8, no. 2: 180–190. 10.1002/rse2.235.

[ece371893-bib-0003] Bird, J. P. , B. K. Woodworth , R. A. Fuller , and J. D. Shaw . 2021. “Uncertainty in Population Estimates: A Meta‐Analysis for Petrels.” Ecological Solutions and Evidence 2, no. 3: e12077. 10.1002/2688-8319.12077.

[ece371893-bib-0004] Bolton, M. 1995. “Food Delivery to Nestling Storm Petrels: Limitation or Regulation?” Functional Ecology 9, no. 2: 161–170. 10.2307/2390560.

[ece371893-bib-0005] Bolton, M. 1996. “Energy Expenditure, Body‐Weight and Foraging Performance of Storm Petrels *Hydrobates pelagicus* Breeding in Artificial Nesting Chambers.” Ibis 138, no. 3: 405–409. 10.1111/j.1474-919X.1996.tb08058.x.

[ece371893-bib-0006] Bolton, M. , J. G. Brown , H. Moncrieff , N. Ratcliffe , and J. D. Okill . 2010. “Playback Re‐Survey and Demographic Modelling Indicate a Substantial Increase in Breeding European Storm‐Petrels *Hydrobates pelagicus* at the Largest UK Colony, Mousa, Shetland.” Seabird 23: 14–24.

[ece371893-bib-0007] Bolton, M. , D. Sheehan , S. E. Bolton , J. A. C. Bolton , and J. R. F. Bolton . 2017. “Resurvey Reveals Arrested Population Growth of the Largest UK Colony of European Storm‐Petrels *Hydrobates pelagicus*, Mousa, Shetland.” Seabird 30: 15–30.

[ece371893-bib-0008] Bolton, M. , M. J. Wood , and O. Padget . 2019. “Stormie Shiny Apps; Shiny Seabirds: duFeu Method (version3).” https://leopet8.wixsite.com/mysite/analyse.

[ece371893-bib-0009] Burnham, K. P. , and D. R. Anderson . 2003. Model Selection and Multimodel Inference: A Practical Information‐Theoretic Approach. Springer Science & Business Media.

[ece371893-bib-0010] Buxton, R. T. , and I. L. Jones . 2012. “Measuring Nocturnal Seabird Activity and Status Using Acoustic Recording Devices: Applications for Island Restoration.” Journal of Field Ornithology 83, no. 1: 47–60. 10.1111/j.1557-9263.2011.00355.x.

[ece371893-bib-0012] Collins, S. M. 2021. “Terrestrial and Marine Risks for Leach's Storm‐Petrels During the Breeding Season.” Vol. PhD. Memorial University of Newfoundland.

[ece371893-bib-0013] Davis, P. 1957. “The Breeding of the Storm Petrel.” British Birds 1, no. 3: 85–101.

[ece371893-bib-0014] Favaro, L. , E. Cresta , O. Friard , et al. 2021. “Passive Acoustic Monitoring of the Endangered African Penguin ( *Spheniscus demersus* ) Using Autonomous Recording Units and Ecoacoustic Indices.” Ibis 163, no. 4: 1472–1480. 10.1111/ibi.12970.

[ece371893-bib-0015] Francomano, D. , A. N. Raya Rey , B. L. Gottesman , and B. C. Pijanowski . 2024. “Acoustic Recording Complements Camera Traps for Monitoring Sensitive Penguin Populations.” Ibis 166, no. 1: 38–54. 10.1111/ibi.13235.

[ece371893-bib-0016] Ghani, B. , T. Denton , S. Kahl , and H. Klinck . 2023. “Global Birdsong Embeddings Enable Superior Transfer Learning for Bioacoustic Classification.” Scientific Reports 13, no. 1: 22876. 10.1038/s41598-023-49989-z. 38129622 PMC10739890

[ece371893-bib-0017] Gladbach, A. , C. Büßer , R. Mundry , and P. Quillfeldt . 2009. “Acoustic Parameters of Begging Calls Indicate Chick Body Condition in Wilson's Storm‐Petrels *Oceanites oceanicus* .” Journal of Ethology 27, no. 2: 267–274. 10.1007/s10164-008-0115-y.

[ece371893-bib-0018] Golightly, R. T. , and S. R. Schneider . 2011. Development of Methods for Bioacoustic Monitoring of Leach's and Fork‐Tailed Storm Petrels at Castle Rock National Wildlife Refuge: 2007–2009 Report. Department of Wildlife, Humboldt State University. https://scholarworks.calstate.edu/downloads/7p88cj852.

[ece371893-bib-0019] Google LLC . 2024. “Google Perch.” https://github.com/google‐research/perch/tree/main.

[ece371893-bib-0020] Google Research . 2023. “Bird‐Vocalization‐Classifier (Online).” Https://Www.Kaggle.Com/Models/Google/Bird‐Vocalization‐Classifier/Frameworks/tensorFlow2/Variations/Bird‐Vocalization‐Classifier/Versions/3.

[ece371893-bib-0021] Hart, K. A. , S. Oppel , G. R. W. Humphries , A. Blackburn , and K.‐B. Nam . 2021. “Estimating Streaked Shearwater *Calonectris leucomelas* Abundance in the Republic of Korea Using Automated Acoustic Recorders.” Marine Ornithology 49: 109–117.

[ece371893-bib-0022] Kassambara, A. 2019. “ggpubr: “ggplot2” Based Publication Ready Plots. R Package Version 0.2.4.” https://CRAN.R‐project.org/package=ggpubr.

[ece371893-bib-0023] Keitt, B. S. 2005. “Status of Xantus's Murrelet and Its Nesting Habitat in Baja California, Mexico.” Marine Ornithology 33: 105–114.

[ece371893-bib-0024] Kelly, K. G. , C. M. Wood , K. McGinn , et al. 2023. “Estimating Population Size for California Spotted Owls and Barred Owls Across the Sierra Nevada Ecosystem With Bioacoustics.” Ecological Indicators 154: 110851. 10.1016/j.ecolind.2023.110851.

[ece371893-bib-0025] Klenova, A. V. 2015. “Chick Begging Calls Reflect Degree of Hunger in Three *Auk* Species (Charadriiformes: Alcidae).” PLoS One 10, no. 11: e0140151. 10.1371/journal.pone.0140151. 26536362 PMC4633236

[ece371893-bib-0026] Linares, C. G. , R. A. Phillips , and R. T. Buxton . 2022. “Monitoring Vocal Activity and Temporal Patterns in Attendance of White‐Chinned Petrels Using Bioacoustics.” Emu ‐ Austral Ornithology 122, no. 1: 27–38. 10.1080/01584197.2021.2018337.

[ece371893-bib-0027] Lisovski, S. , S. Bauer , M. Briedis , et al. 2020. “Light‐Level Geolocator Analyses: A User's Guide.” Journal of Animal Ecology 89, no. 1: 221–236. 10.1111/1365-2656.13036. 31190329

[ece371893-bib-0028] Minguez, E. , and D. Oro . 2003. “Variations in Nest Mortality in the European Storm Petrel *Hydrobates pelagicus* .” Ardea 91, no. 1: 113–117.

[ece371893-bib-0029] Oliver, R. Y. , D. P. W. Ellis , H. E. Chmura , et al. 2018. “Eavesdropping on the Arctic: Automated Bioacoustics Reveal Dynamics in Songbird Breeding Phenology.” Science Advances 4, no. 6: eaaq1084. 10.1126/sciadv.aaq1084. 29938220 PMC6010323

[ece371893-bib-0030] Oppel, S. , S. Hervias , N. Oliveira , et al. 2014. “Estimating Population Size of a Nocturnal Burrow‐Nesting Seabird Using Acoustic Monitoring and Habitat Mapping.” Nature Conservation 7: 1–13. 10.3897/natureconservation.7.6890.

[ece371893-bib-0031] Pérez‐Granados, C. , and J. Traba . 2021. “Estimating Bird Density Using Passive Acoustic Monitoring: A Review of Methods and Suggestions for Further Research.” Ibis 163: 765–783. 10.1111/ibi.12944.

[ece371893-bib-0032] Quillfeldt, P. 2002. “Begging in the Absence of Sibling Competition in Wilson's Storm‐Petrels, *Oceanites oceanicus* .” Animal Behaviour 64, no. 4: 579–587. 10.1006/anbe.2002.3090.

[ece371893-bib-0033] Quillfeldt, P. , and J. F. Masello . 2004. “Context‐Dependent Honest Begging in Cory's Shearwaters (*Calonectris diomedea*): Influence of Food Availability.” Acta Ethologica 7, no. 2: 73–80. 10.1007/s10211-004-0100-6.

[ece371893-bib-0034] R Core Team . 2023. R: A Language and Environment for Statistical Computing. R Foundation for Statistical Computing. http://www.R‐project.org/.

[ece371893-bib-0035] Raine, A. F. , M. Boone , M. McKown , and N. Holmes . 2017. “The Breeding Phenology and Distribution of the Band‐Rumped Storm Petrel *Oceanodroma castro* on Kaua'i and Lehua Islet, Hawaiian Islands.” Marine Ornithology 45: 73–82.

[ece371893-bib-0036] Ratcliffe, N. , D. Vaughan , C. Whyte , and M. Shepherd . 1998. “Development of Playback Census Methods for Storm Petrels *Hydrobates pelagicus* .” Bird Study 45, no. 3: 302–312. 10.1080/00063659809461101.

[ece371893-bib-0037] Ricklefs, R. E. 1992. “The Roles of Parent and Chick in Determining Feeding Rates in Leach's Storm‐Petrel.” Animal Behaviour 43, no. 6: 895–906. 10.1016/S0003-3472(06)80003-5.

[ece371893-bib-0038] Robb, M. , K. Mullarney , and The Sound Approach . 2008. Petrels Night and Day. Sound Approach. https://soundapproach.co.uk/product/petrels‐night‐day/.

[ece371893-bib-0039] Schackwitz, W. , D. A. Airola , A. Greene , M. Schackwitz , and J. Woodruff . 2020. “Bioacoustic Monitoring Reveals Details of Tricolored Blackbird Breeding Phenology.” Journal of Fish and Wildlife Management 11, no. 2: 518–530. 10.3996/102019-JFWM-083.

[ece371893-bib-0040] Scott, D. 1970. “The Breeding Biology of the Storm Petrel *Hydrobates pelagicus*.” PhD. University of Oxford In Edward Grey Institute. https://ora.ox.ac.uk/objects/uuid:24973c21‐ab78‐42f1‐a3c1‐a7665b776bbf.

[ece371893-bib-0041] Soanes, L. M. , R. J. Thomas , and M. Bolton . 2012. “Evaluation of Field and Analytical Methods for Estimating the Population Size of Burrow‐Nesting Seabirds From Playback Surveys.” Bird Study 59, no. 3: 353–357. 10.1080/00063657.2012.695334.

[ece371893-bib-0042] Stretcu, O. , E. Vendrow , K. Hata , et al. 2023. “Agile Modeling: From Concept to Classifier in Minutes.” *arXiv*. 10.48550/arXiv.2302.12948.

[ece371893-bib-0043] Sutherland, D. R. , and P. Dann . 2012. “Improving the Accuracy of Population Size Estimates for Burrow‐Nesting Seabirds.” Ibis 154, no. 3: 488–498. 10.1111/j.1474-919X.2012.01234.x.

[ece371893-bib-0044] Walsh, P. , D. Halley , M. P. Harris , A. del Nevo , and I. Sim . 1995. “Seabird Monitoring Handbook for Britain and Ireland.” Tasker ML Peterborough: JNCC/RSPB/ITE/Seabird Group. http://nora.nerc.ac.uk/8798/1/Bird1.pdf.

[ece371893-bib-0045] Watanuki, Y. 1986. “Moonlight Avoidance Behavior in Leach's Storm‐Petrels as a Defense Against Slaty‐Backed Gulls.” Auk 103, no. 1: 14–22. 10.1093/auk/103.1.14.

[ece371893-bib-0046] Winkler, D. W. , S. M. Billerman , and I. J. Lovette . 2020. “Shearwaters and Petrels (Procellariidae), Version 1.0.” Birds of the World. 10.2173/bow.procel3.01species_shared.bow.project_name.

[ece371893-bib-0047] Wood, S. N. 2011. “Fast Stable Restricted Maximum Likelihood and Marginal Likelihood Estimation of Semiparametric Generalized Linear Models.” Journal of the Royal Statistical Society 73, no. 1: 3–36.

