# Supplementary material for: Bioacoustics as a Measure of Population Size and Breeding Success of European Storm Petrels Hydrobates pelagicus
Source: Ecol Evol. 2025 Aug 11;15(8):e71893. doi: 10.1002/ece3.71893 (PMC12336414; doi:10.1002/ece3.71893)
Supplement: Supplementary file 1 — Data S1. [file ECE3-15-e71893-s001.zip › DataS1.docx]

**Supplementary Material:**

**Supplementary Material 1 Identifying AudioMoth lateral detection range**

Experimental set up and data processing


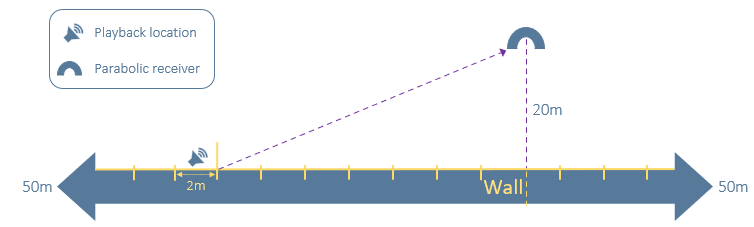
To identify the range over which storm petrel calls may be expected to be detected by the receivers we undertook a series of detection tests. For the detection tests we played a recording of storm petrel calls twice at 2m intervals along the length of each plot, as per Supplementary Figure 1.

**Supplementary Figure 1 A schematic diagram of playback detection tests. Dotted arrows indicate distances measured with a laser range finder.**

Each detection test consisted of 6s of adult-purr, 6s adult-chat and 6s chick call (18 seconds in total). We included a pure tone in the detection test audio to clearly distinguish between each 2m section in recordings. We played calls at the same volume of unsolicited storm petrels calls recorded in the colony during this study (~75 dB). We also measured the direct line distance at each point to the receiver in each plot using a laser range finder, accurate to the nearest meter. We measured these direct line distances to also assess how the absolute distance from a receiver to the playback position may affect audibility.

We undertook all detection range tests during daylight hours to reduce any disturbance on storm petrel activity in the colony, as this almost entirely takes place at night. Following these tests, we noted when receivers detected calls at each of the distances along the plot by playing back the recordings from each AudioMoth at full volume. Each recording was listened to and scored by two observers. If the two observer’s scores did not agree, then the recording for that plot and distance were listened to by a third observer who determined whether the call was truly audible or not (adult-purr: n= 62/930 plot distances, 6.67%; adult-chat: n= 55/930, 5.59%; chick = 40/930, 4.3%).

*Statistical analysis*

We used GLMMs to determine the lateral detection range of the three storm petrel call types. We modelled the ‘audbility’ of calls as a binary response of ‘0’ inaudible or ‘1’ audible with a logit link. We included the ‘distance of playback along the wall’ and the direct line ‘receiver distance’ as continuous variables and ‘call type’ as a three-level unordered factor. We included a three way interaction term between the explanatory variables to assess how the relative audibility of the call types changed in relation to the distance they were played from the receiver and their distance along the wall. ‘Plot’ was included as a random effect.

Ultimately, we determined a call to be no longer reliably audible when the likelihood of audibility was below 50%.

*Results:*

Across call types audibility decreased as the distance along the wall increased (estimate= -0.17, 95%CI= -0.24, -0.11; Supplementary Table 1).

**Supplementary Table 1 Outputs from a GLMM assessing the effect of storm petrel call type (adult: purr, adult: chat, and chick:) and distance of call playback along a wall on audibility of calls. Significant terms i.e., those with 95% confidence intervals not overlapping zero, are in bold. Call type ‘adult: chat’ was used as a reference level.**

| ***Fixed effects:*** | **Estimate** | **Standard error** | **95% confidence interval** |
| --- | --- | --- | --- |
| ***Intercept*** | **5.31** | **0.67** | **4.01, 6.77** |
| ***Distance along wall (m)*** | **-0.17** | **0.03** | **-0.24, -0.11** |
| *Call type- adult: purr* | -0.58 | 0.54 | -1.67, 0.48 |
| ***Call type- chick*** | **-1.19** | **0.54** | **-2.29, -0.12** |
| *Distance along wall (m) * Call type- adult: purr* | -0.004 | 0.02 | -0.04, 0.03 |
| ***Distance along wall (m) * Call type- chick*** | **-0.09** | **0.02** | **-0.14, -0.05** |
|  |  |  |  |
| *Marginal R^2^= 0.53, Conditional R^2^= 0.85, n= 1395 observations* | | | |

Chick calls were significantly less audible than chat calls (estimate: -1.19, 95%CI= -2.29, -0.12, Supplementary Table 1). However, there was no difference between the audbibility of chat and purr calls (estimate= -0.58, 95%CI= -1.67, 0.48, Supplementary Table 1). The rate of decline in audibility of chick calls with increasing distance along the wall was also greater than that for chat and purr calls (estimate= -0.09, 95%CI= -0.14, -0.05, Supplementary Table 1 and Supplementary Figure 2). There was no difference in the rate of decline of audibility with increasing distance between chat and purr calls (estimate= -0.004, 95%CI= -0.04, 0.03, Supplementary Table 1 and Supplementary Figure 2).

**Supplementary Figure 2 GLMM model predictions of the relationship between storm petrel call type and the distance of the playback of call types along a wall on the audibility of calls. A distance of ‘0’ indicates that the call was played directly opposite the recorder.**


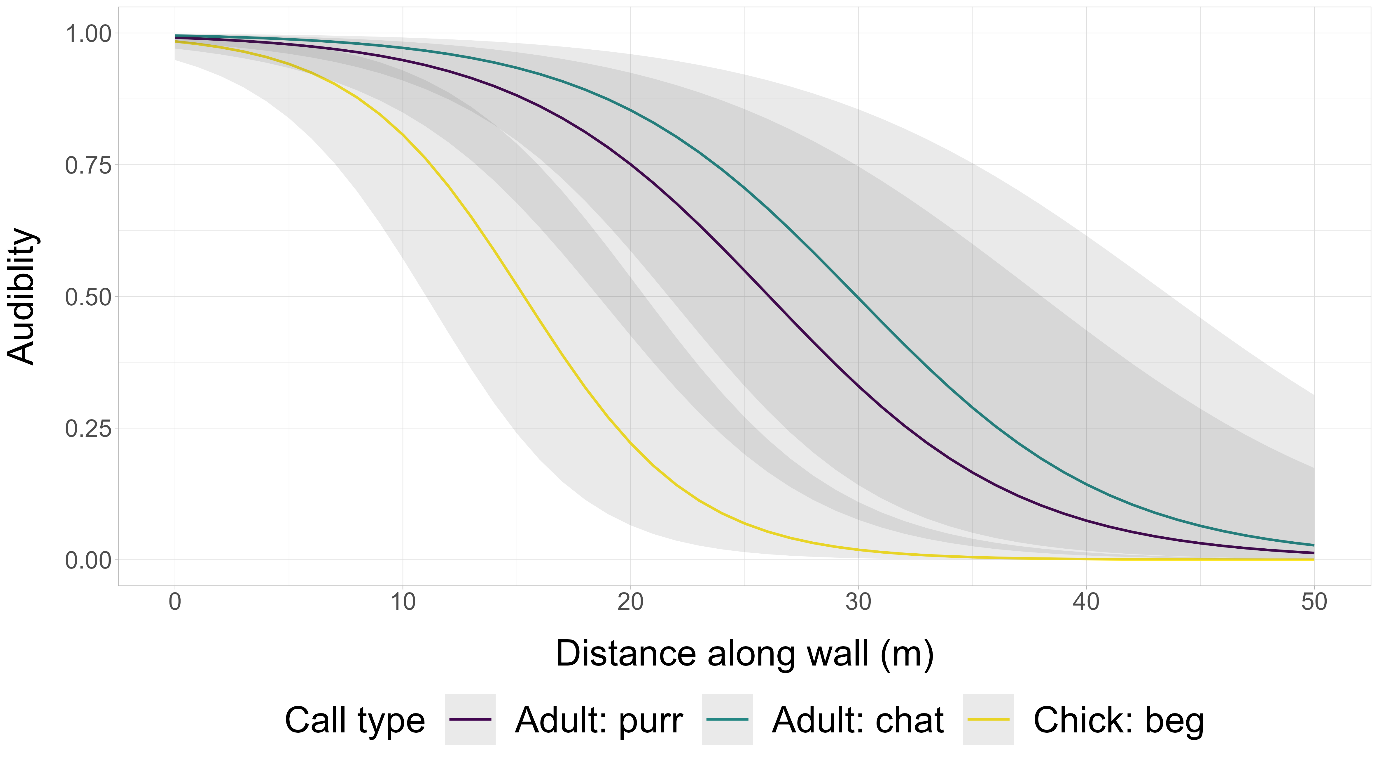


Considering calls to no longer be reliably audible when the likelihood of audibility was lower than 0.5, adult-purr calls were no longer reliably audible beyond 26m (95%CI= 18m- 38m), adult- chat calls beyond 30m (95%CI= 22-42) and chicks calls 16m (95%CI= 10-20).

**Supplementary Material 2 Bioacoustic recording periods**

Prior to any analysis of the bioacoustic data we first filtered out all ‘unusable’ data files, i.e., those where excessive background noise from wind would prevent storm petrel calls from being reliably heard, Supplementary Table 2.

**Supplementary Table 2 The bioacoustic recording periods for each plot for adult and (chick) calls. For some nights there was partial usable/ unusable data.**

| **Plot** | **Start date** | **End date** | **Number of survey nights** | | |
| --- | --- | --- | --- | --- | --- |
|  |  |  | *With usable data* | *With unusable data* | *Missing data* |
| 1 | 08/06/2023 | 26/08/2023 | 26 (21) | 13 (18) | 0 (0) |
| 2 | 09/06/2023 | 26/08/2023 | 26 (22) | 12 (25) | 0 (0) |
| 3 | 09/06/2023 | 28/08/2023 | 23 (21) | 10 (8) | 3 (8) |
| 4 | 09/06/2023 | 26/08/2023 | 28 (32) | 9 (13) | 0 (0) |
| 5 | 09/06/2023 | 26/08/2023 | 32 (18) | 3 (8) | 0 (0) |
| 6 | 09/06/2023 | 26/08/2023 | 36 (15) | 1 (1) | 0 (0) |
| 7 | 09/06/2023 | 26/08/2023 | 33 (29) | 6 (9) | 0 (0) |
| 8 | 09/06/2023 | 26/08/2023 | 28 (7) | 8 (13) | 0 (0) |
| 9 | 09/06/2023 | 26/08/2023 | 32 (14) | 6 (9) | 0 (0) |
| 10 | 09/06/2023 | 26/08/2023 | 29 (13) | 8 (13) | 0 (0) |
|  | **Total** | | 291 (192) | 77 (117) | 3 (8) |

Those files with excessive background noise from wind were characterised by frequent periods with a minimum amplitude of less than -125 dB. We determined unusable files to be those where the number of seconds where the minimum amplitude was less than -125 dB accounted for more than 20% of the seconds in a file. At this chosen threshold there was 100% agreement between the automated assessment of file quality and observer-based judgement by SB using a random sample of 400 files.

We concurrently recorded the calls of chicks in nestboxes around the plots, Supplementary Table 3.

**Supplementary Table 3 The bioacoustic recording periods for each nestbox**

| **Nestbox** | **Estimated hatch date of chick** | **End date** | **Number of survey days** | | |
| --- | --- | --- | --- | --- | --- |
|  |  |  | *With usable data* | *With unusable data* | *Missing data* |
| 1 | 27/07/2023 | 27/08/2023 | 32 | 0 | 0 |
| 2 | NA | NA | NA | NA | NA |
| 3 | 27/07/2023 | 27/08/2023 | 32 | 0 | 0 |
| 4 | 30/08/2023 | 27/08/2023 | 3 | 0 | 26 |
| 5 | 20/07/2023 | 27/08/2023 | 39 | 0 | 0 |
| 6 | 20/07/2023 | 27/08/2023 | 39 | 0 | 0 |
| 7 | 29/07/2023 | 27/08/2023 | 30 | 0 | 0 |
| 8 | 26/07/2023 | 27/08/2023 | 33 | 0 | 0 |
| 9 | 26/07/2023 | 27/08/2023 | 33 | 0 | 0 |
| 10 | 23/07/2023 | 27/08/2023 | 36 | 0 | 0 |
|  | **Total** | | 277 | 0 | 26 |

Nestbox 2 failed prior to the chick stage, hence no data for this nestbox are included in the above table.

**Supplementary Material 3 Classification and quantification of call rates**

We used an active learning technique called Agile Modelling (Monarch, 2021; Stretcu et al., 2023). Agile Modelling takes advantage of a pre-trained deep learning model that is exposed to large amounts of labelled data, which is useful even if the model has not seen the exact classes or data relevant for the task at hand. Agile modelling requires a comparatively small number of labelled audio data for target calls to achieve reliable classification than e.g., deep learning based audio classification. Consequently, as little labelled audio data exists for the call types of interest, and to prevent a great time investment in labelling new audio, we used Agile modelling. To adapt the model to new classes, Agile Modelling leverages representations created by the later layers of the pre-trained model that are called ‘embeddings’, a set of abstract vectors that the model uses to undertake classification. The other key part of Agile Modelling is a human-in-the-loop data labelling process to efficiently create a new, custom model based on those embeddings. Because we are utilising the embeddings generated by the large pre-trained model, the bespoke model is much simpler and can be trained much more efficiently than a full from-scratch deep learning model. The custom model we use is a simple linear classifier, and we only need a small number of initial examples (5 used for both adult calls and 8 for chick calls) and several rounds of labelling to produce a high-quality classifier.

Specifically, we use a pre-trained bird vocalisation classifier model (Google Research, 2023), which is based on an EfficientNet-B1 architecture (Tan & Le, 2019) and is trained on weakly-labelled audio from Xeno-canto (Xeno-canto Foundation and Naturalis Biodiversity Center, 2005) a public bird recording repository. We refer the reader to Hamer et al (2023) for more details.

Before the human-in-the-loop labelling process commences, we use the pre-trained network to embed all non-overlapping 5 second segments of a large, unlabelled audio corpus. The output gives an embedding for each segment; each embedding has as lower dimensionality than the original raw audio clip, and the set of such embeddings is semantically richer to search over.

To start the labelling process, we use the same pre-trained model to generate embeddings for the known audio clips for each target label. Generating the embeddings yields a set of vectors in embedding space for which we know the correct label. The active learning system then finds and returns a set of k nearest neighbours (kNN) drawn from the embedded unlabelled corpus using the standard Euclidean metric, i.e., comparing the known query to every other embedding in the unlabelled corpus. The matching neighbour vectors are then returned to the user along with its corresponding audio segment (a 5 second clip from the unlabelled dataset). The user then listens to these audio clips and annotates them with the correct labels, bootstrapping a corpus of labelled examples from the large, unlabelled dataset.

After generating sufficient examples for each of the target classes, we can train a simple linear classifier model over the embeddings corresponding to the bootstrapped labelled examples. In the model the explanatory variable was the (embedded) 5-second snippet of raw audio. This explanatory variable was a single 1280-dimensional vector representing the embedded 5-second audio sample.  The linear model then takes as input this 1280-dimensional vector, and returns a sequence of logits (i.e., non-normalized values representing "probability"), one logit for each of the target classes, which in our case were 'chick', 'purr', and 'chat', and also an 'unknown' class. The model then predicts that the audio is a 'chat' if the logit score for 'chat' is greater than the threshold value, which was the response. We used a Binary Cross-Entropy loss, which is akin to a binary classifier on each class. The new linear model can then be used to find another set of k nearest audio segments, which in turn can be relabelled to further refine the model. The process can be repeated until the user is satisfied with this classifier model. Prior work has shown that this technique is both sample efficient and highly effective in the domain of labelling bioacoustics data according to standard audio classification metrics like AUC-ROC (Ghani et al., 2023).

To make our model for European Storm Petrels, *Hydrobates pelagicus*, we wished to label four new sound classes: ’adult: purr’, ‘adult: chat’, and ‘chick’, 'unknown', where ‘unknown’ represents any other sound. Using the procedure described above, we labelled 'N’ examples over ‘M’ labelling iterations (‘N’ for adult: purr= 18, adult: chat= 16, chick= 12, and unknown= 8, ‘M’ for all call types= 3).  The linear model was then trained on 5 randomly chosen samples from each class.  Resulting model quality was then evaluated on the whole labelled dataset.  Our model yielded a final score of 0.91 for cmAP, 0.84 for AUC-ROC, and 0.89 for Top-1, indicating a high confidence in classifications of all types.

**Supplementary Material 4 Location of nest boxes fitted with audiomoths**

***
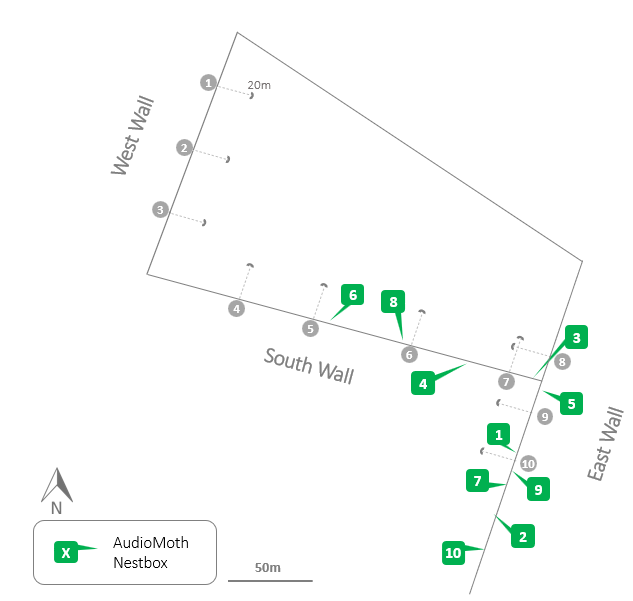
***The relative locations of all AudioMoths placed in nestboxes can be seen in Supplementary Figure 3.

**Supplementary Figure 3 The location of nest boxes fitted with an AudioMoth in the study area. Nest boxes are labelled with a unique ID. The location of plots and receivers are provided for context.**

**Supplementary Material 5 Quantifying the required number of survey days to reach an consistent measure of call rate**

*Methods*

Nightly adult call rates are subject to variation that may arise from bird behaviour, environmental effects and combinations of the two (Buxton & Jones, 2012; Watanuki, 1986). Consequently, call rates across a large sample of nights are required to obtain average measures of call rates that are independent of behavioural and environmental variation (Buxton & Jones, 2012). The arising average call rate then takes into account the potential variation in call rates that may result in biased call rates when estimated over insufficiently long periods of time. Accordingly, prior to undertaking the main analysis we quantified whether we had sufficient nights of data to reach a stable measure of call rate using similar methods those used by [Buxton & Jones (2012)](#_ENREF_4) in Leach’s storm petrels.

For the two adult call types (adult: purr and adult: chat) and the chick call type, we calculated average nightly call rates in each plot, varying the sample size from which means were calculated from one night up to the maximum number of nights of usable data available for each plot. The nights included in each sample were selected randomly and with replacement. We randomised the selection of nights up to a total of 36 or 43 days (the total length of the potential recording period for adults and chicks respectively). We repeated the randomisation procedure 1000 times for each plot. We then estimated the number of nights of data required for the curve to reach a plateau in each iteration, i.e., when any increase in the number of nights included in the sample did not results in any clear change in the average nightly call rate. We determined a plateau to be reach when the slope of the curve fell below 0.1 ([Buxton & Jones 2012](#_ENREF_4)). We then calculated the average number of days need to reach a plateau for each plot and adult call type across the 1000 repetitions.

*Results*

For both adult call types in all plots a plateau in the mean nightly call rate was reached within the number of days with data available, Supplementary Table 4. Consequently, we have a sufficient number of days of data to obtain stable call rates for adults. Were a stable call rate expected for chicks, then this assumption would not have been met however.

**Supplementary Table 4 The average number of days with data (+ standard deviation) required to reach a plateau in the mean nightly call rate in each plot for each call type.**

| **Plot** | **Call type** | | |
| --- | --- | --- | --- |
|  | *Adult: purr* | *Adult: chat* | *Chick* |
| 1 | 12 | 25 | 25 |
| 2 | 11 | 8 | 19 |
| 3 | 9 | 17 | 14 |
| 4 | 8 | 4 | 29 |
| 5 | 10 | 7 | 33 |
| 6 | 26 | 6 | 7 |
| 7 | 30 | 30 | 34 |
| 8 | 6 | 6 | 24 |
| 9 | 7 | 3 | 31 |
| 10 | 11 | 13 | 14 |
| **Average** | 13 +8.18 | 11.9 +9.29 | 25+9.19 |
